# Long-Term Care Providers’ Perspectives on Health Information Exchange During Patient Transitions Into Long-Term Care: A Multiple Case Study

**DOI:** 10.1177/08404704261426444

**Published:** 2026-02-22

**Authors:** Augustine Chukwuebuka Okoh, Aimun Shah, Christine Lin, Paranshi Gupta, Naisha Dharia, Caroline Caswell, Henry Yu-Hin Siu, Michelle Howard, Amit Arya, Holly Odoardi, Brian McKenna, Keya Shah, Sarah Wojkowski, Lawrence Grierson

**Affiliations:** 1 Department of Health Research Methods, Evidence and Impact, 3710 McMaster University, Hamilton, Ontario, Canada; 2 Department of Family Medicine, 3710 McMaster University, Hamilton, Ontario, Canada; 3 Department of Family and Community Medicine, 7938 University of Toronto, Toronto, Ontario, Canada; 4 Department of Healthy and Safe Communities, RinggoldID:10001 City of Hamilton, Hamilton, Ontario, Canada; 5 School of Rehabilitation Science, 3710 McMaster University, Hamilton, Ontario, Canada; 6 MERIT Research Centre, 3710 McMaster University, Hamilton, Ontario, Canada

## Abstract

This multiple case study explored how Long-Term Care (LTC) teams in Ontario, Canada, manage informational continuity when older adults transition from community-based care to LTC. Five LTC homes, varying in size and rurality, participated, with 20 professionals interviewed across various roles, including nursing, medicine, rehabilitation, and administration. LTC providers emphasized the importance of comprehensive, accurate, and up-to-date biopsychosocial information to support effective care. However, information transferred from community and hospital sources was often incomplete or outdated. To address gaps, LTC staff sought additional details from electronic health records, families, care coordinators, and hospitalists. Their ability to obtain missing information was influenced by organizational capacity, physician’s practice location, power dynamics between providers, availability of family caregiver support, and access to electronic health records. A stronger primary/hospital-LTC collaboration, incentives for informational continuity, and a specific staff managing transition information and activities could optimize the LTC transition information exchange process.

## Introduction

The typical Long-Term Care (LTC) resident has cognitive impairment, difficulty ambulating, incontinence, and chronic multi-morbidities.^1^ They require around the clock nursing, personal care, and supervision. When they enter LTC, they lose contact with their community primary care provider, and the home’s interprofessional team of nurses, nurse practitioners, social workers, personal support workers, and physicians assume responsibility.^2,3^ Relational discontinuity during LTC transitions remains unabated in Ontario given the system challenges collaboration across primary care and LTC settings.^4^ Thus, the probability of retaining one’s previous family physician after moving into LTC is low, with available evidence showing that nearly 90% of Ontarian seniors who move into LTC experience relational discontinuity.^3^ This disruption increases the chances of medical errors, rehospitalization, and mortality.^5^ Accordingly, homes rely on informational continuity to mitigate the negative impact of relational discontinuity during LTC transitions. Informational continuity refers to the efficient flow of patient health information across the continuum of care. It involves using comprehensive information about a patient’s preferences, values, context, conditions, and personal circumstances to support optimal care delivery.^6^

In Ontario, LTC homes receive two main documents during LTC transition: the Long-Term Care Health Assessment Form (LTC-HAF), which contains the primary care provider’s medical report, and the Resident Assessment Instrument-Minimum Data Set (RAI-MDS), which is completed by a care coordinator from the regional health authority and contains assessments of the new residents’ psychological, social, and physical functioning.^7^ However, as a point of reference, a recent American study indicated that the incompleteness and untimeliness of the information provided by the patient-discharging care providers via similar jurisdictionally relevant forms stands as a substantial limitation in supporting older adults’ LTC transitions.^8^ Through a large nationally representative survey, the investigators found that information related to functional, mental, and behavioural status and follow-ups were missing in the forms transmitted in almost two-thirds of transition-to-LTC cases.^8^ Similar patterns are likely to occur in Ontario, Canada, as well.

While numerous studies^4,9,10^ have affirmed the utility of informational continuity as a viable solution to address disrupted relational continuity, they also highlight that much remains to be empirically understood on how LTC providers engage with the information exchange process during LTC transitions, and the kinds of information they deem important to maintain good-quality care for LTC residents. Hence, this study sets out to describe the information LTC care teams consider to be most important to support informational continuity during community to LTC transitions, the information they receive and do not receive, the strategies they employ to seek out missing information, and factors influencing their ability to seek out the information.

## Methods

### Research Design

**Figure 1.**
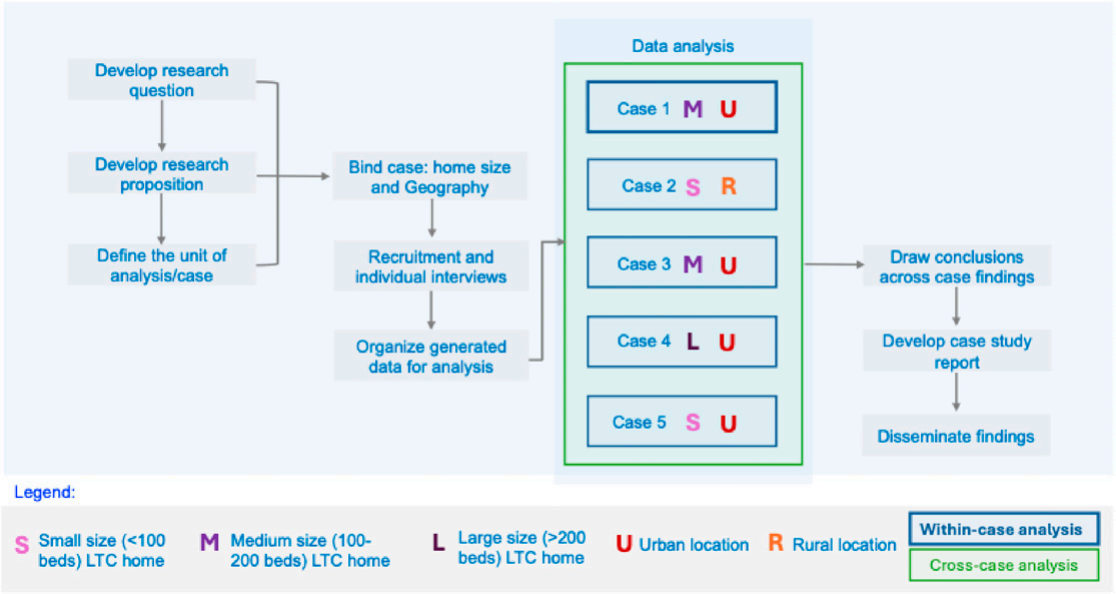
Overview of the research methods

Multiple case study (Figure 1).^11,12^

### Cases

Ontario-based LTC homes were purposively selected for variation in rurality and size. Case recruitment was facilitated through partners with the Greater Hamilton Health Network and the Ontario Long Term Care Clinicians.

### Participants

Eligible participants were care providers at each case who play a role in assessments and information exchange during intake processes.

### Data Collection

Primary data were collected via semi-structured participant interviews between October and December 2024. Interviews probed the information providers receive during LTC transitions, the kind of information they would like to receive, and their sources of relevant information during the transitions. They also sought the participants’ perceptions of facilitators and barriers to informational care continuity in their context. Interviews were conducted in person, audio-recorded, transcribed, and de-identified prior to analysis.

### Data Analysis

We adopted an unconstrained deductive approach to content analysis,^13^ which was informed by Transitions Theory.^14^ We began within-case analyses before moving to between-case analysis. Analysis concluded upon data sufficiency.^15^

### Ethics Approval

This study was approved by the Hamilton Integrated Research Ethics Board (#16209).

## Results

Five LTC homes, comprising 1 rural and 4 urban homes, participated in the study. As shown in Table 1, the cases were 1 large, 2 medium, and 2 small bed capacity homes. Twenty in-depth interviews were completed from participants across the 5 cases. Participants were aged 36-63 years and their years of LTC practice experience ranged from 1-40 years. Thirteen identified as women and 7 identified as men. Participants held a variety of professional roles including admission nurse, director of care, physiotherapist, admission coordinator, social worker, personal support worker, pharmacist, nurse practitioner, and LTC physician.

**Table 1.** Description of Case Profiles and Participant Characteristics

Features	Case 1	Case 2	Case 3	Case 4	Case 5
Case profile					
Home size (beds)	192	47	167	350	96
Rurality	Urban	Rural	Urban	Urban	Urban
Ownership status	Non-profit	For-profit	Non-profit	Non-profit	For-profit
Participant characteristics (n = 20)					
Gender					
Men	-	-	4	1	2
Women	4	3	3	1	2
Average years of LTC practice	22.8	9.7	14.1	21.3	18.3
Care provider roles	Director of care, social worker, admission nurse, admission coordinator	Director of care, admission nurse, admission coordinator	Physician, director of care, admission nurse, physiotherapist, personal support worker, pharmacist	Physician, social worker	Nurse practitioner, director of care, assistant director of care, social worker, admission nurse

### Information Valued

With respect to the information participants deemed important, they stated that past medical and surgical histories, medication lists, functional abilities, immunization records, and recent blood work often contained in the LTC-HAF are valuable to them. They also value the psychosocial and physical function related information, usually contained in the RAI-MDS. The new LTC resident’s psychosocial information, delineating their personal care needs, values, and their likes and dislikes, was deemed important in determining the room to assign them, the amount of care they need, and guide the personalization of care (Table 2, quote 1). In this regard, all participants declared person-centred care as a cardinal value underpinning their aspiration for informational continuity during LTC transitions.

**Table 2.** Representative Quotes

Quote no.	Quotes
1	“Their likes and dislikes are important” … “personalize care plans” … “Our hope is that residents’ daily routine aligns with their preferences.” (C3-P14)
2	“Sometimes people are not as forthcoming because they know that putting all the information in the application, people may reject them and say, ‘No way, we don’t want to admit this person.’ So, sometimes people are very open, honest, and transparent and sometimes people are not, and there are discrepancies between what we see on paper versus the person when they come.” (C4-P09)
3	“You’re looking at something that may have changed… we have files updated the last 3 months, but things may have changed in a week.” (C4-P12)
4	“Sometimes the information is very accurate and very detailed, and sometimes it isn’t… Some people will take more time to fill it out and give a lot of details. For some people, you can just tell they’re just checking it off and not putting in as much.” (C4-P09)
5	“Often, we’ll just depend on the families themselves; we might say, bring us what you had at home. We often get a medication list from the previous pharmacist. It’s easier for me to call a pharmacist than to get something from the physician.” (C2-P05)
6	“I’ve never really talked to the family doctors. You just ask their secretary to get information; requests like the chart, and they send the chart over.” (C3-P08)
7	“Being a small home, most of us wear different hats. We don’t have an IPAC [Infection prevention and control] person, so she [Director of care] is doing some IPAC work. We’re stretched thin… That’s what we do here with our home size. Whereas in bigger homes, you might see all those positions filled. Actually, they do. So, they are able to do more during admission.” (C2-P07)
8	“[H]e had his family practice here for years. He also worked in the ER at our local hospital. So, everybody knew him; all the doctors know each other. He was able to coordinate much better because he knew everybody.” (C2-P06)
9	“I would probably more likely [have] the nurse practitioner [make the request], because once they say they’re the nurse practitioner, they attract more attention. Coming from me [a nurse], it doesn’t really hold enough weight. It’s a shame that it has to be like that. It shouldn’t have to be like that.” (C1-P02)
10	“A lot of people’s families aren’t involved sometimes, and some people don’t have families.” (C5-P20)

### Information Received and Not Received

Common across all cases, participants affirmed that a comprehensively filled out LTC-HAF and RAI-MDS were useful for the success of their work in LTC. The typical information they receive includes the patient’s medical and surgical history, x-rays, bloodwork, infections, medication list, lifestyle assessment (e.g., drinking and smoking), behaviour (e.g., wandering), incontinence and mobility status, activities of daily living needs, and the use of assistive devices like wheelchairs, glasses, dentures, and hearing aids. Participants talked about some additional documents they occasionally receive in support of informational continuity. These include documents related to comprehensive behavioural assessments for individuals with behavioural and psychological symptoms of dementia, notes for any surgeries, and notes from the previous interdisciplinary care team (e.g., dietitian’s assessment and physiotherapist’s assessment) for those transitioning from the hospital.

Although participants acknowledged that the aforementioned pieces of information were useful, they reported that the information may be lacking in detail or not cover the breadth of information needed in LTC. While an up-to-date immunization record was said to be important, it is often missing from the LTC-HAF. Also, they shared that they rarely receive information concerning functional abilities, advance care plans, and the frequency and type of any pertinent oxygen delivery system (e.g., tank, concentrator, nasal prongs, and masks). They also encountered situations where important information such as diagnosis (e.g., psoriasis) or critical details of patient history was missing in the admission package. Furthermore, the participants expressed concerns that the LTC-HAF and RAI-MDS may not contain the most reliable, comprehensive, and up-to-date information of the patient’s current condition. For one, the LTC home sometimes receives documents with incomplete records, which was attributed to the current lack of a minimum standard for volume of information or details that are required in the documents. This provides a leeway for certain information (e.g., for patients with aggressive behaviours) to be left out purposely (quote 2). Also, the last assessment relative to the time of LTC admission might have occurred months earlier, with any changes occurring during that period not captured in the documents (quote 3). Sometimes, the information received may be less detailed because of a lack of attention to detail (quote 4).

### Strategies Employed to Fill the Information Gap

While participants rely on the documents (LTC-HAF and RAI-MDS) for patient care transitions, they often need to employ other strategies to get the information they desire. When they have access to a harmonized hospital Electronic Health Record (EHR), they will usually start by referring to it for more comprehensive information. However, where such a resource is not present or the information therein is unsatisfactory, they typically resort to talking to families and occasionally to contacting care coordinators, hospitalists, and/or community pharmacists, for any up-to-date or missing information they need. These partners-in-care were described as more accessible than discharging physicians, whether in community or hospital (quote 5). Indeed, participants indicated that communication between discharging family physicians and LTC physicians or other LTC staff was vanishingly rare (quote 6).

### Factors Affecting LTC Providers’ Ability to Seek Out More Information

While participants reported that they could reach out to a resident’s family or other care providers or search harmonized EHRs in order to fill information gaps, our data also revealed five factors that could influence their ability to implement those strategies.

#### Capacity

Participants described capacity issues related to time and staffing constraints. Generally, participants said that LTC providers hardly reach out to or initiate communication with the discharging care providers mainly because of work pressure and a short, 5-day admission window. Considering that these care providers already have significant workloads to deal with, the short window was said to be too little to coordinate inter-provider interactions for each new LTC intake. A LTC home’s staffing capacity could influence overall workload and their willingness to pursue additional information. This issue was predominant in the smaller, rural LTC home, which has lesser resources and staff than the larger, urban LTC homes. This LTC home reported difficulty recruiting and retaining staff in the rural setting. Hence, staff members take up multiple roles to cover gaps (quote 7).

#### Physician’s Practice Location

The LTC physician’s practice location also influences their information seeking behaviour during LTC transitions. In the rural case, for instance, we heard that their previous LTC physician lived and had a primary care practice locally. Being embedded in the rural community, this physician had close professional relationships with other local care providers; hence, they could easily reach out to those providers for needed patient information. However, their current LTC physician now only visits on designated *“doctor days”* from another city. Accordingly, no relationships have been established between the physician and the local primary and acute care providers that can facilitate information gathering. This was typical of the relationships in the 4 urban cases, where the LTC physician rarely interacted with the discharging care providers. Embeddedness in a tight rural healthcare environment seemed to promote the type of relationships that promote inter-physician information exchange (quote 8).

#### Professional Power Dynamics

Perceptions of professional power imbalance influenced information seeking behaviours. Participants recounted instances of reaching out to discharging care providers for more information and noting biases in the likelihood of receiving a response based on the professional identity of the staff who sent the information request (quote 9).

#### Lack of Family Caregiver Support

LTC providers interact with families and rely on them for some of their information needs. Participants noted that families can serve as intermediaries between the LTC care teams and the discharging care providers. However, the family’s level of involvement in the older adult’s care determines how much information they can facilitate (quote 10). When this involvement is low or absent, the LTC providers are less likely to seek the family’s input.

#### Access to an EHR

Participants reported differences in their ability to access EHRs. The LTC providers who do not have access to hospital or primary care physicians’ EHRs are unable to use these tools to supplement information deficiencies.

## Discussion and Implications for Health Leaders

Faced with relational discontinuity, LTC providers would rely on informational continuity at key points of transition in support of quality care delivery. Thus, the study findings underscore several important implications for health leaders related in relation to clinical practice, education, and policy, offering actionable recommendations to support informational continuity during LTC transitions. For clinical practice, the findings highlight the integration of direct cross-setting provider communications as a normative activity during the LTC admission process. This communication could enhance the quality and accuracy of information transferred. It could foster greater practitioner engagement in the information-sharing process and reduce the risk of omission or loss of important information during care transitions, thereby promoting continuity and patient safety.

Health professions education systems can also be leveraged to attune care providers to appreciate the significance of comprehensive information transfer.^4^ In this regard, it would be pertinent to ensure focused content on LTC transitions in the core curricula of health professions training programs. This learning material should address the logistical aspects of transitioning care while also emphasizing the broader implications for continuity of care. Interprofessional education that highlights collaborative practice, effective communication strategies, and the import of the informational continuity dimensions of transitions in care will better prepare healthcare professionals to manage these complex processes.^16^

From a policy perspective, strategic investment in the LTC transition workforce is needed, particularly through the creation of a dedicated LTC role to support the systematic collection of comprehensive information during admissions. It would reduce the workload and relieve pressure on LTC staff currently involved in both direct or indirect resident care and admissions. This would mitigate burnout but also allows LTC care transition staff the time and capacity to engage in activities that could produce informational continuity, such as seeking out any additional information from various sources.

Collectively, these recommendations centre a system-level approach to improving LTC transitions and align with the Integrated People-Centred Health Systems (IPCHS) standard, particularly coordination across the care continuum and optimization of access, flow, and transitions.^17^ In principle, the IPCHS standard supports inter-provider communication across settings, accessible health information systems, and seamless patient information flow. Ultimately, the health system in Ontario (and other jurisdictions) should move beyond siloed structures. Setting standards for individual sectors (long-term care, hospitals, primary care, and home care) is no longer sufficient when patient care routinely crosses organizational and professional boundaries. Siloed standards do little to prevent dangerous care gaps when residents move between settings. For instance, data from the United States shows that poor transitions are linked to serious safety risks, contributing to 80% of serious medical errors and nearly $20 billion annually in preventable healthcare cost.^18^ Thus, true quality and safety depend on how well LTC, hospitals, and community-based services work together as one system. Ontario must, therefore, set a clear expectation that integration across sectors is not optional, but central. Standards should explicitly require shared accountability, interoperable information systems, and coordinated communication, ensuring that patients experience continuity of care rather than fragmentation as they move through the health system.

## Conclusion

LTC providers value informational continuity during LTC transitions, deeming it crucial for a person-centred approach to care to optimize residents’ care outcomes and quality of life. However, they often lack the enabling conditions to practice in ways that support informational continuity. Improving their ability to seek out more information may require stronger cross-setting collaboration between community and LTC providers, incentives for services that promote informational continuity, and the establishment of a designated LTC role to coordinate transition information and activities, allowing other LTC staff to focus on other administrative and care responsibilities. Regarding limitation, the study focused on the experiences of care providers without incorporating the perspectives of older adults and their families in this informational continuity discourse. In future work, an inquiry into the experiences and perceptions of older adults and their family caregivers on the information-sharing tools and process and their implications for informational continuity-related policies and practice would be desirable.

## Data Availability

The dataset generated and analyzed during the current study is not publicly available because individuals’ privacy could be compromised, but it is available from the corresponding author upon reasonable request.*
